# Quality control in SARS-CoV-2 RBD-Fc vaccine production using LC–MS to confirm strain selection and detect contaminations from other strains

**DOI:** 10.1038/s41598-024-59860-4

**Published:** 2024-04-26

**Authors:** Pipob Suwanchaikasem, Kaewta Rattanapisit, Richard Strasser, Waranyoo Phoolcharoen

**Affiliations:** 1 Baiya Phytopharm Co., Ltd., Bangkok, 10330 Thailand; 2 Department of Applied Genetics and Cell Biology, Institute of Plant Biotechnology and Cell Biology, University of Natural Resources and Life Sciences, 1180 Vienna, Austria; 3 Center of Excellence in Plant-Produced Pharmaceuticals, Chulalongkorn University, Bangkok, 10330 Thailand; 4 Department of Pharmacognosy and Pharmaceutical Botany, Faculty of Pharmaceutical Sciences, Chulalongkorn University, Bangkok, 10330 Thailand

**Keywords:** Biological techniques, Biotechnology, Drug discovery

## Abstract

Coronavirus disease of 2019 (COVID-19), caused by severe acute respiratory syndrome coronavirus 2 (SARS-CoV-2), is an ongoing outbreak, disrupting human life worldwide. Vaccine development was prioritized to obtain a biological substance for combating the viral pathogen and lessening disease severity. In vaccine production, biological origin and relevant materials must be carefully examined for potential contaminants in conformity with good manufacturing practice. Due to fast mutation, several SARS-CoV-2 variants and sublineages have been identified. Currently, most of COVID-19 vaccines are developed based on the protein sequence of the Wuhan wild type strain. New vaccines specific for emerging SARS-CoV-2 strains are continuously needed to tackle the incessant evolution of the virus. Therefore, in vaccine development and production, a reliable method to identify the nature of subunit vaccines is required to avoid cross-contamination. In this study, liquid chromatography-mass spectrometry using quadrupole-time of flight along with tryptic digestion was developed for distinguishing protein materials derived from different SARS-CoV-2 strains. After analyzing the recombinantly produced receptor-binding domain (RBD) of the SARS-CoV-2 spike protein, nine characteristic peptides were identified with acceptable limits of detection. They can be used together to distinguish 14 SARS-CoV-2 strains, except Kappa and Epsilon. Plant-produced RBD-Fc protein derived from Omicron strains can be easily distinguished from the others with 4–5 unique peptides. Eventually, a peptide key was developed based on the nine peptides, offering a prompt and precise flowchart to facilitate SARS-CoV-2 strain identification in COVID-19 vaccine manufacturing.

## Introduction

Coronavirus disease of 2019 (COVID-19), emerging in 2019 from Wuhan city, Hubei province, China, has threatened human health and life globally. More than 6.9 million deaths have been recorded since the beginning of the pandemic^1^. Severe acute respiratory syndrome coronavirus 2 (SARS-CoV-2) is a causative pathogen of the disease. Although drugs for treating SARS-CoV-2, such as remdesivir, molnupiravir and nirmatrelvir, have been developed, their clinical achievements have not been well documented and potential side effects need long-term monitoring^2–4^. Preventive methods, such as hand sanitization, wearing face masks and social distancing are still first-line strategies to prevent disease spreading. Immunization with COVID-19 vaccines is a recommended regime, which is highly effective to reduce disease severity and mortality upon infection^5^. It is effective due to its known mechanism that pre-introduction of attenuated or death virus into our body can teach the immune cells to memorize pathogen patterns and be prepared for live pathogens^6,7^. Nowadays, several types of COVID-19 vaccine, such as mRNA, protein subunit and viral vector vaccines, have been successfully developed and used. Nonetheless, continuation in COVID-19 vaccine research is necessary to catch up with the emergence of new SARS-CoV-2 strains^8–10^.

Baiya Phytopharm Co., Ltd. (https://baiyaphytopharm.com/) is a Thai university-based company, aiming to develop COVID-19 vaccines for Thai population, ensuring COVID-19 vaccine access to local people. Its success will help reducing burden from international vaccine support and preventing global vaccine shortage. A plant-manufactured approach is applied for recombinant protein production, where *Nicotiana benthamiana* is used as a manufacturing plant^11,12^. The vaccine product is designed by merging the receptor-binding domain (RBD) region of SARS-CoV-2 with the fragment crystallizable (Fc) region of human immunoglobulin 1 (IgG1)^13,14^. RBD is a key fragment of the SARS-CoV-2 spike that interacts with angiotensin-converting enzyme 2 (ACE2) receptor on host cell surface membrane to allow viral penetration into human body^15^. SARS-CoV-2 constantly mutates the RBD region to avoid memorization by human immune system and enhance the binding to ACE2. To date, a number of mutation sites on RBD regions have been identified, resulting in a variety of SARS-CoV2 strains, for example Wuhan wild-type, Alpha, Beta, Gamma, Delta, Epsilon, Zeta, Eta, Theta, Iota, Kappa, Lambda, Mu and Omicron^16^. The prototype of SARS-CoV-2 RBD-Fc vaccine has been successfully developed using the Wuhan RBD sequence. The final vaccine product is formed by conjugating the recombinant RBD-Fc protein with alum adjuvant. Wuhan RBD-Fc vaccine has reached phase II clinical trial, whereas the other SARS-CoV-2 vaccines are being developed^17^.

During vaccine production, quality control (QC) is an important step to verify product attributes^18,19^. This step is required for our production process to confirm the authenticity of RBD-Fc derived from Wuhan SARS-CoV-2 strain and to detect contaminants that could arise from the RBD-Fc products of other SARS-CoV-2 strains. Liquid chromatography-mass spectrometry (LC–MS) is a reliable technique for determining protein sequences to confirm protein identity^20^. Its application in protein identification and quantification has been expanded due to its high sensitivity and robustness^21^. It has been successfully used to identify SARS-CoV-2 strains in biological samples including saliva and nasal swabs^22–24^, making it a desirable analytical platform for examining SARS-CoV-2-related substances in biologic production.

In this study, LC–MS was applied to confirm the recombinant RBD-Fc protein derived from Wuhan SARS-CoV-2 strain and to detect protein contaminants derived from other SAR-CoV-2 strains. Wuhan wild type and other 13 SARS-CoV-2 variances were included in this analysis. After tryptic digestion, nine characteristic peptides were identified from the tandem MS results and validated for limit of detection. They can be cooperatively used to identify RBD regions of different SARS-CoV-2 strains. The developed method has consolidated the QC process, enabling systematic assessment on intermediate substances and final vaccine products.

## Results

### Analysis of different SARS-CoV-2 RBD-Fc protein sequences

**Figure 1:**
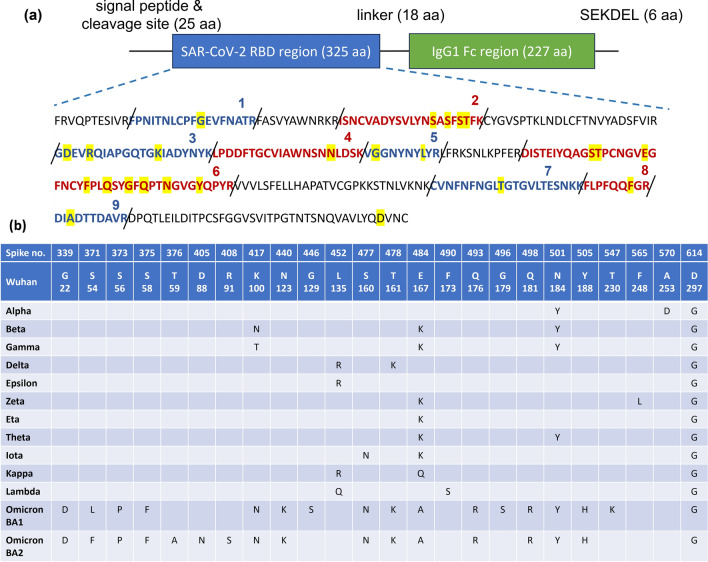
Amino acid differences of RBD region among all 14 SARS-CoV-2 strains included in this study. (**a**) Protein construct of SARS-CoV-2 RBD-Fc IgG1 fusion with magnification of amino acid sequences along the RBD region, showing nine characteristic peptides obtained upon trypsin digestion. Amino acid changes are highlighted in yellow. (**b**) Details of amino acid changes at RBD region of 13 SARS-CoV-2 strains as compared to Wuhan strain. “Spike no.” refers to the corresponding position in a full-length sequence of Wuhan SARS-CoV-2 spike glycoprotein.

In total, 14 SARS-CoV-2 strains including Wuhan wild-type and other 13 strains, i.e., Alpha, Beta, Gamma, Delta, Epsilon, Zeta, Eta, Theta, Iota, Kappa, Lambda, Omicron BA1 and Omicron BA2 were analyzed in this study. Based on protein sequence, several point mutations are observed among RBD sequences of all 14 strains (Fig. 1). Omicron BA1 and BA2 are the most distinguished strains, where 17 amino acids of their RBD regions are different from that of Wuhan wild type. While Epsilon and Eta strains show only 2 amino acids different from Wuhan strain (Fig. 1b). Based on LC–MS/MS analysis, nine characteristic peptides were identified from the resulting MS spectrum (Fig. 1a). These peptides contain at least one variable amino acid among all strains and can be used together to identify SARS-CoV-2 strains in the tested samples.

### Confirmation of recombinant Wuhan SARS-CoV-2 RBD-Fc protein

**Figure 2:**
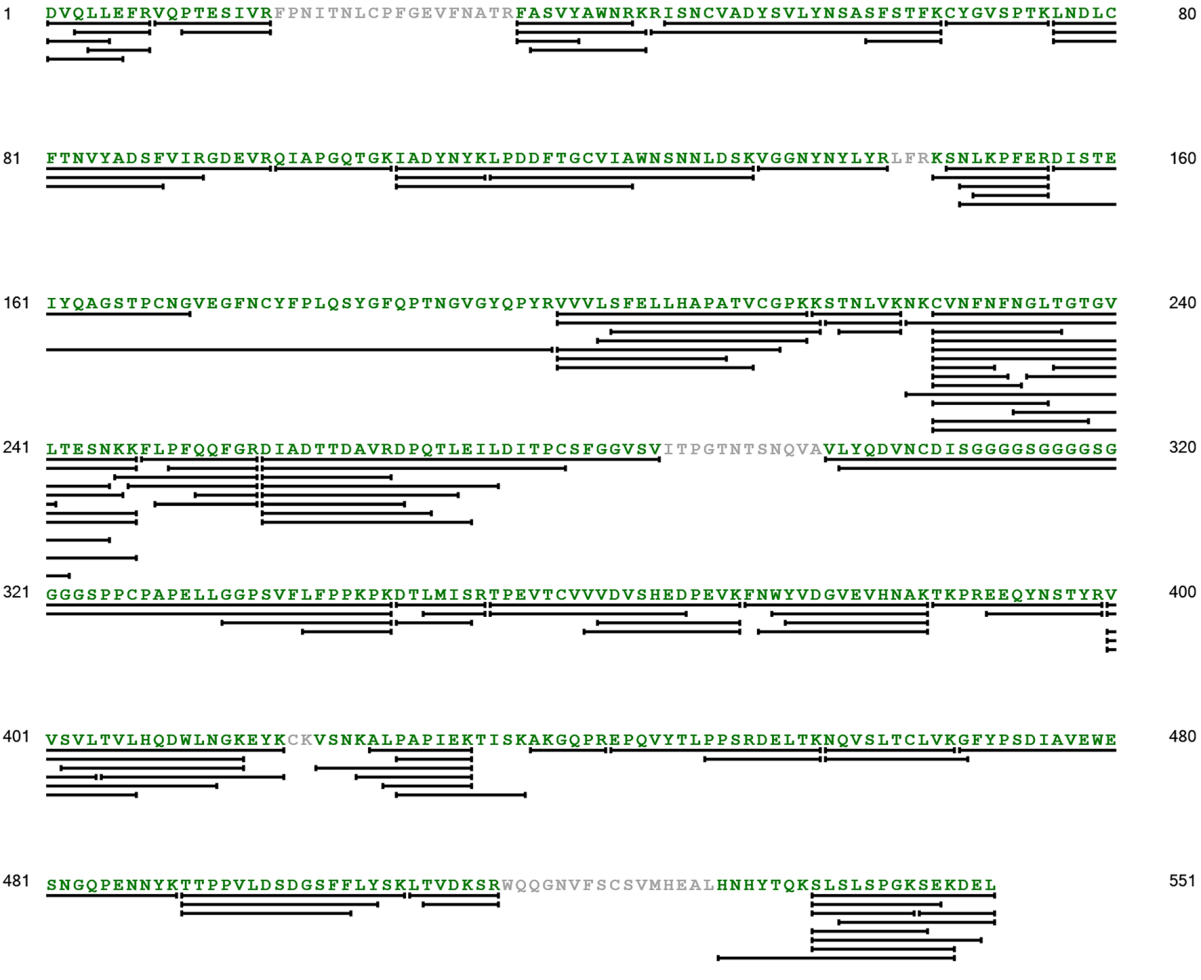
Confirmation of Wuhan RBD-Fc IgG1 protein by LC–MS/MS data. Peptides detected with MS/MS fragmentation are highlighted in green. Black lines under amino acid sequences demonstrate coverage of each peptide. Overall sequence coverage of this sample was 90.74%, which twos of the three missing peptides were potentially glycosylated.

SARS-CoV-2 RBD-Fc protein derived from Wuhan strain was confirmed with tandems MS data, showing 90.74% sequence coverage (Fig. 2), where the detected peptides with MS/MS fragmentation are bolded in green. Twos of the three missing peptides contain glycosylated sites (NXS or NXT), likely forming high-mannose glycopeptides. Therefore, their unmodified forms were hardly detected in LC–MS/MS analysis. Apart from peptide 1, eight of nine characteristic peptides were matched to the reference Wuhan sequence at MS/MS level. Within similar settings, peptide 1 was sometimes detected with MS/MS fragmentation, but the MS/MS data were missing in some other peptides, such as peptide 5 and peptide 6, instead (Supplementary Fig. S1). Raw LC chromatogram and MS/MS spectrum of the representative peptides are displayed in Supplementary Fig. S2.

### Identification of characteristic peptides among different SARS-CoV-2 RBD-Fc proteins

**Figure 3:**
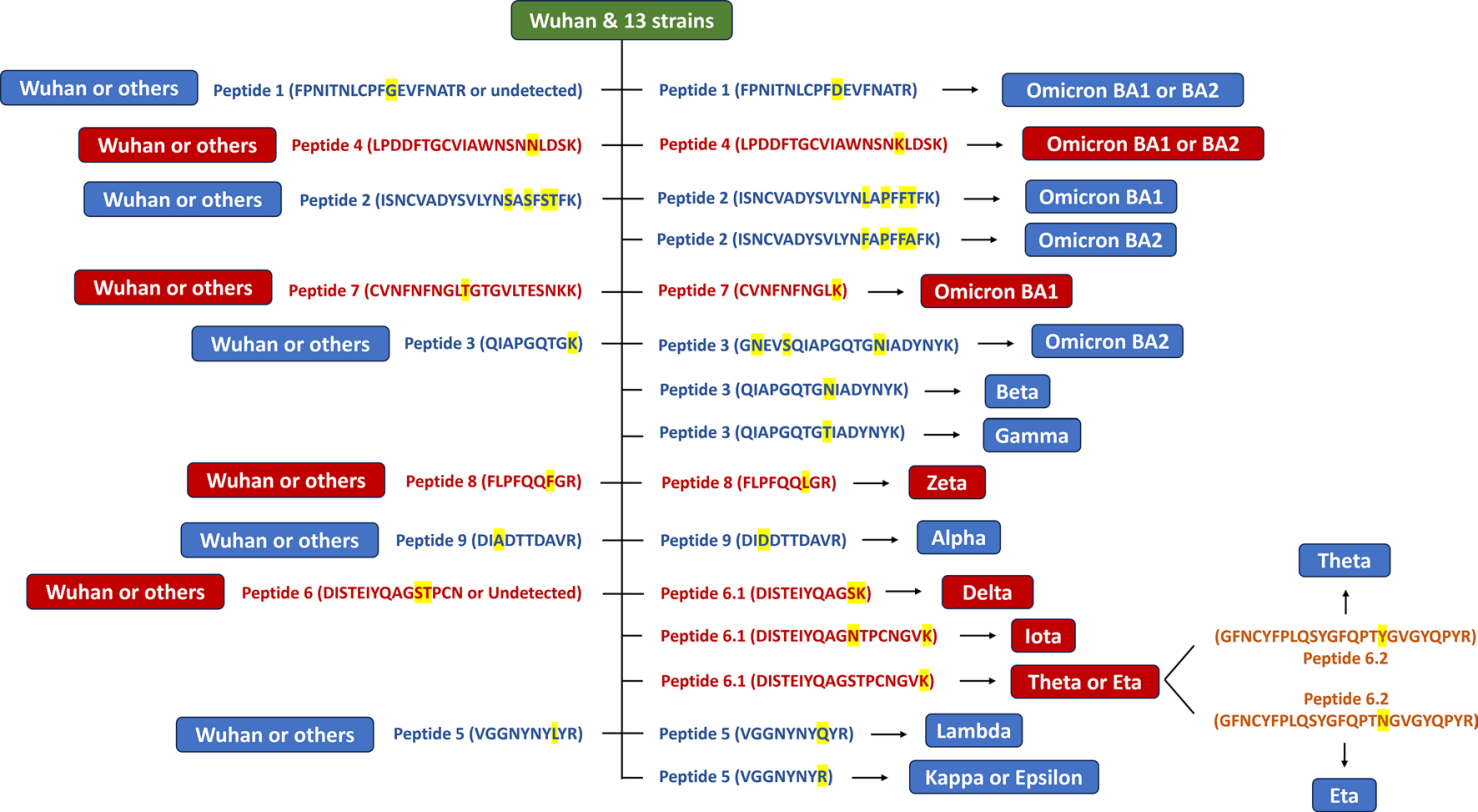
Peptide key with nine characteristic peptides for identification of SARS-CoV-2 strains. The identification process flows from the top to the bottom. Left side of the flowchart shows peptide common for Wuhan and other strains, whereas right side of the flowchart shows peptide unique for particular SARS-CoV-2 strains.

Resulting peptides of other SARS-CoV-2 strains were detected with multiple variations of amino acids as compared to Wuhan wild type. For example, peptide 2 of Omicron BA1 showed variations of S54L, S56P and S58F positions, while peptide 2 of Omicron BA2 was identified with S54F, S56P, S58F and T59A different from Wuhan strain. This peptide can be used for distinguishing Wuhan, Omicron BA1 and BA2 strains. In some cases, the peptides of SARS-CoV-2 variants exactly matched to that of Wuhan strain. For example, peptides 8 of Wuhan and Omicron BA1 and BA2 strains were FLPFQQFGR. Hence, this peptide cannot be used to differentiate Omicron strains from Wuhan wild type. However, peptide 8 with FLPFQQLGR sequence was unique for Zeta strain and, therefore, can be used to distinguish Zeta strain from the others. The details of all nine characteristic peptides are presented in Supplementary Table S1. Based on these selected peptides, a peptide key was generated to facilitate protein identification and differentiation (Fig. 3).

### Peptide key

Peptide key (Fig. 3) was designed as a stepwise flowchart. Upon use, it is needed to follow in order from the top to the bottom, where highly distinct and easily distinguishable strains, such as Omicron, Beta, Gamma and Zeta strains, are listed on the top. Peptides 1 and 4 are characteristic for Omicron strains. Their sequences between Omicron BA1 and BA2 are identical but different from the other strains. Therefore, they can be used to distinguish Omicron BA1 and BA2 strains from the others. Other peptides (peptide 2, 3, 5, 6, 7, 8 and 9) are unique for certain SARS-CoV-2 strains. Peptide 7 with CVNFNFNGLK sequence is a unique peptide for Omicron BA1. Peptides 3 with the sequences of GNEVSQIAPGQTGNIADYNYK, QIAPGQTGNIADYNYK and QIAPGQTGTIADYNYK are unique for Omicron BA2, Beta and Gamma strains, respectively. Peptides 8 and 9 are unique for Zeta and Alpha strains, respectively. Peptide 5 is unique for Lambda strain with L135Q substitution. It is also applicable for separating Kappa and Epsilon strains from the others according to L135R substitution. However, Kappa and Epsilon cannot be distinguished from each other due to one-amino-acid difference. At position 167, glutamic acid (E) represents Epsilon strain and glutamine (Q) belongs to Kappa strain. This position is located on peptide 6. By nature, this peptide is hardly detected by LC–MS analysis due to high hydrophobicity and lengthy sequence (43 amino acids). Its MS and MS/MS signals were relatively low as compared to the other peptides (Supplementary Fig. S2). Therefore, Kappa and Epsilon strains cannot be differentiated by the developed LC–MS technique. Nonetheless, peptide 6 of certain SARS-CoV-2 strains was cleavable by trypsin due to lysine (K) substitution at T161 or E167 sequence, yielding characteristic peptides for Delta and Iota strains. Cleaved peptide 6 (peptide 6.2) can be used to differentiate Theta and Eta strains from each other.

### Application of the peptide key to identify RBD-Fc proteins from different SARS-CoV-2 strains

**Figure 4:**
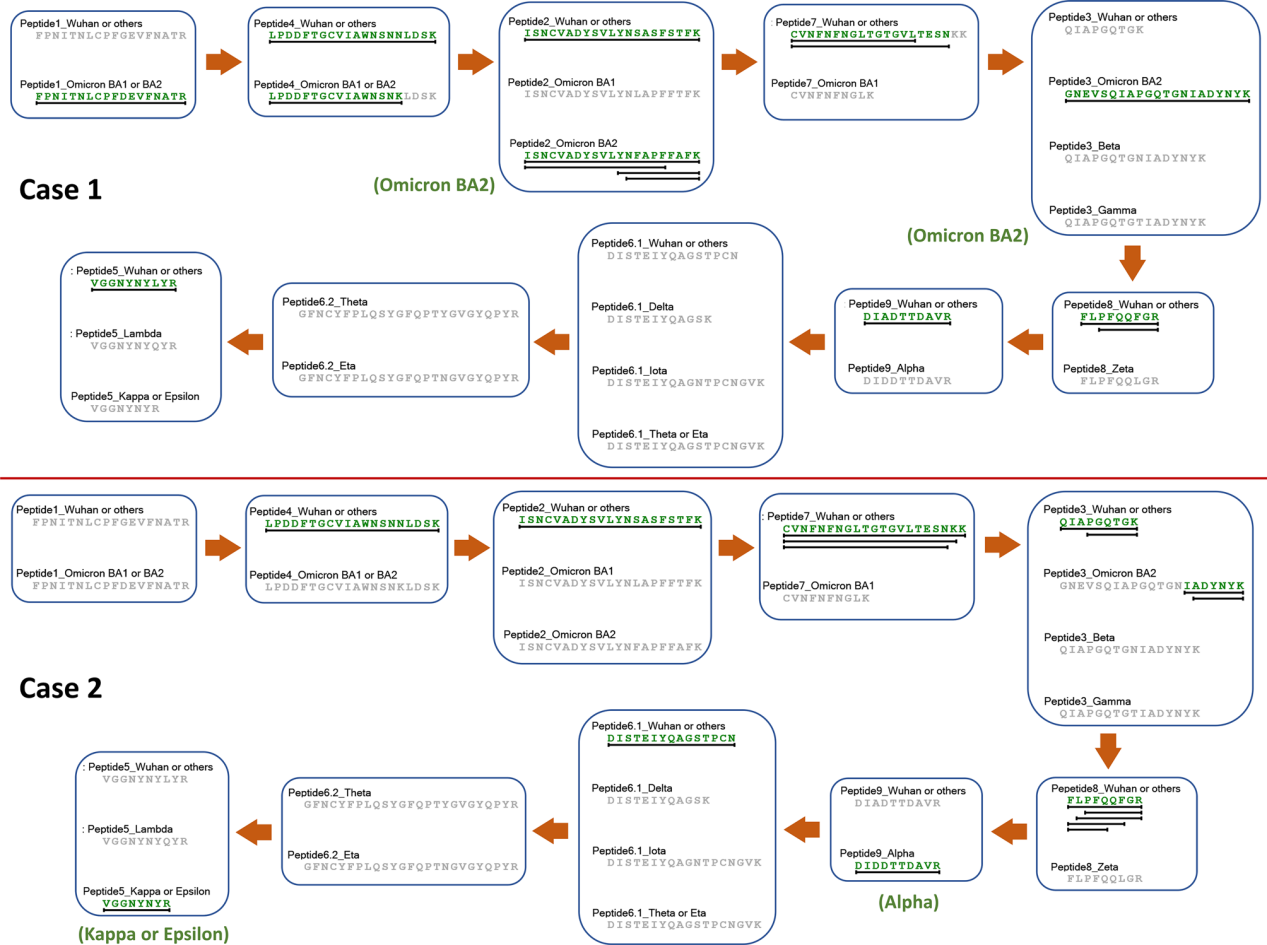
Examples showing applications of the peptide key to identify RBD-Fc proteins derived from different SARS-CoV-2 strains. In the top panel (case 1), Omicron BA2 was mainly identified, along with partial sequences of Wuhan strain. In the bottom panel (case 2), either Alpha and Kappa or Alpha and Epsilon were two SARS-CoV-2 strains mixed in this RBD-Fc sample.

Examples showing the stepwise identification of recombinant RBD-Fc proteins derived from different SARS-CoV-2 strains according to the nine characteristic peptides are shown in Fig. 4. In the top panel (case 1), Omicron BA2 strain was identified from the sample. Unique peptides 2 and 3 of Omicron BA2 were clearly detected. Peptides 1 and 4, common peptides for Omicron strains, were also found. In addition, peptides 2 and 4 of Wuhan strain were concomitantly detected, indicating that Wuhan strain might be mixed with Omicron BA2 in this sample. In the bottom panel (case 2), peptide 9 unique for Alpha strain and peptide 5 unique for Kappa or Epsilon strains were observed. In contrast, peptides 5 and 9 common for the other strains were not simultaneously detected. Unique peptides of the other strains were neither observed, implying that this sample could be a mixture of Alpha and Kappa strains or Alpha and Epsilon strains.

### Detection limit of potential contaminants in RBD-Fc protein

The detection limit of all nine characteristic peptides is displayed in Table 1. The lowest protein concentrations, where all peptides were detected in MS and MS/MS modes, were 0.5 and 5 ng ml^−1^, respectively. By using the formula, limits of detection ranged between 4.58 to 113.37 ng ml^−1^. The limit of peptide 6.1 detection was not determined since at least 100 ng ml^−1^ is required to obtain the signal of this peptide. Peptides 5 and 8 were the most sensitive peptides with the lowest concentrations of 5–10 ng ml^−1^ detected at MS/MS level, implying that contaminants of RBD-Fc proteins from Lambda, Kappa or Epsilon and Zeta strains can be delicately detected. Detections of Gamma strain with peptide 3 and Iota strain with peptide 6.1 were relatively sensitive at MS/MS level. Moreover, peptide 3 for Beta strain, peptide 4 for Omicron BA1 and BA2 strains, peptide 7 for Omicron BA1 strain and peptide 9 for Alpha strain showed relatively low limit of detection in MS analyzing mode as compared to MS/MS mode, suggesting that their fragmentations were tentatively low and MS data would be helpful for identifying these SARS-CoV-2 strains.

**Table 1:** Detection limit of nine characteristic peptides.

Peptide no	Sequence	SARS-CoV-2 strain	m/z	Lowest concentration detected with MS/MS (ng ml^−1^)	Lowest concentration detected with MS only (ng ml^−1^)	Limit of detection based on MS calibration curve (ng ml^−1^)
1	FPNITNLCPFDEVFNATR	Omicron BA1 or BA2	1078.0187	200	10	113.37
2	ISNCVADYSVLYNLAPFFTFK	Omicron BA1	823.7428	50	10	44.62
	ISNCVADYSVLYNFAPFFAFK	Omicron BA2	825.0682	200	10	84.19
3	GNEVSQIAPGQTGNIADYNYK	Omicron BA2	1120.0373	100	5	53.16
	QIAPGQTGNIADYNYK	Beta	876.9307	200	2	7.35
	QIAPGQTGTIADYNYK	Gamma	870.4331	20	1	9.26
4	LPDDFTGCVIAWNSNKLDSK	Omicron BA1 or BA2	1133.5240	200	2	4.58
5	VGGNYNYQYR	Lambda	617.2857	5	0.5	13.65
	VGGNYNYR	Kappa or Epsilon	471.7250	10	0.5	19.38
6.1	DISTEIYQAGSK	Delta	656.3247	200	100	-
	DISTEIYQAGNTPCNGVK	Iota	656.3112	20	2	13.42
6.2	GFNCYFPLQSYGFQPTYGVGYQPYR	Theta	1003.7938	100	5	39.08
	GFNCYFPLQSYGFQPTNGVGYQPYR	Eta	987.4539	50	5	31.50
7	CVNFNFNGLK	Omicron BA1	606.7947	100	5	5.06
8	FLPFQQLGR	Zeta	553.3122	5	1	5.45
9	DIDDTTDAVR	Alpha	560.7593	200	2	13.08

## Discussion

In vaccine production, QC is an important step to confirm identities of raw materials, relevant ingredients and final products. They are also screened for unwanted contaminants within the QC processes^25,26^. In case of SARS-CoV-2 vaccine production, the presence of other viral strains in the drug substances and/or end products would lead to failure of product tests and may cause a drop in efficacy and/or increase the risk of adverse events^27^. Therefore, establishing a suitable method for checking authenticity of the SARS-CoV-2 strain selected for vaccine production and detecting contaminants from other strains is essential for vaccine quality assessment. LC–MS technique is widely known for its sensitivity, robustness, accuracy and high throughput^28^. It has a benefit over an enzyme-linked immunosorbent assay (ELISA) because it can detect several peptides from one or multiple viral strains in a single run^29^. LC–MS method has been broadly used in clinical research to identify SARS-CoV-2 variants, but surprisingly its application in manufacturing process of biological products has less been found^30^. This study demonstrated that LC–MS can be a useful tool for evaluating product quality in biopharmaceutical production.

Wuhan and early developed SAR-CoV-2 strains, for example Alpha, Beta, Gamma, Delta and Epsilon show little variation among their RBD amino acid sequences, demonstrating their close relationships. However, more recently emerging Omicron strains show 17 amino acids different from Wuhan wild type, indicating substantial evolution of Omicron lineages (Fig. 1). All distinct amino acids detected among 14 SARS-CoV-2 strains contribute to nine characteristic peptides upon tryptic digestion. Most of the characteristic peptides can be readily detected in LC–MS analysis but unmodified peptide 6 is hardly detected. The sequence between 150D and 192R positions (43 amino acids in length) has neither arginine (R) nor lysine (K), digestive sites of trypsin enzyme, causing a lengthy peptide after digestion. The large peptide could become low hydrophilicity, poor ionization, limited fragmentation and could comprise multiple charge states, resulting in low or no signal in LC–MS analysis^31,32^. In the RBD-Fc sequences of different SARS-CoV-2 strains, including Delta, Iota, Theta and Eta strains, T161 or E167 position is substituted with K, yielding digestible peptides, which are useful for differentiating these strains from the others and from each other (Fig. 3). Peptides 1, 2, 3, 4 and 7 are applicable for detecting Omicron BA1 and BA2. Peptide 3 is helpful for identifying Beta and Gamma strains. Peptides 8 and 9 are required for identifying Zeta and Alpha strains, respectively. Peptide 5 is unique for Lambda strain and applicable for distinguishing Kappa and Epsilon strains from the others. When all characteristic peptides were used together (Fig. 3) and the identification processes were followed step by step from the top to the bottom, identification of SAR-CoV-2 strains can be achieved in a single attempt, even from a mix of recombinant RBD-Fc proteins derived from different viral strains (Fig. 4).

However, Kappa and Epsilon strains cannot be differentiated from each other with this LC–MS method because they have only one amino-acid difference, which does not contribute to a characteristic peptide after tryptic digestion. To overcome this issue, GluC protease could be an alternative enzyme for using alone or together with trypsin enzyme to additionally cleave the proteins at C-terminus of glutamic acid (E). GluC enzyme could facilitate the differentiation of SARS-CoV-2 RBD-Fc proteins derived from Epsilon and Kappa strain because the sequence of Epsilon strain contains glutamic acid at position 167 (E167), which is cleavable by GluC enzyme. However, this amino acid is mutated to glutamine (Q167) for Kappa strain. After GluC digestion, the resulting peptides would be different between the samples derived from Epsilon and Kappa strains and therefore distinguishable by LC–MS analysis. Apart from GluC enzyme, other proteases, such as LysC, ArgC and AspN enzymes, could be additionally applied to increase completeness of protein digestion at lysine (K) and arginine (R) positions or additionally cleave the peptide at aspartic acid (D) position to increase number of resulting peptides. This could lead to better detection and differentiation of vaccine products derived from closely related SARS-CoV-2 strains. However, time and cost would be factors of concern as increased number of enzymes will double digestion time and chemical use.

Post-translational modifications of *N*-glycosylation could be another factor, affecting LC–MS results and analytical performance to confirm protein identity and detect contaminations. In this study, characteristic peptide 1 has one glycosylation site at N26 amino acid. Sometimes, MS/MS signal of the non-glycosylated form was not observed. Peptide 1 is important for differentiating RBD-Fc proteins derived from Omicron BA1 and BA2 from the others. To improve the MS/MS signal of peptide 1 and abate the effects of *N*-glycosylation, protease enzymes, such as Endo-S and PNGase A, could be additionally used to release *N*-glycans from the core protein structure. Nonetheless, other characteristic peptides, including peptides 2, 3, 4 and 7, are appliable for distinguishing Omicron BA1 and BA2 strains from each other and from the others. Hence, Endo-S and PNGase A enzymes might not be necessary in this study, but they would benefit the other studies, discovering that only *N*-glycosylated peptide is a characteristic peptide.

In typical LC–MS analysis, the resulting peptides are matched to the reference sequence of individual proteins. For example, the result from Wuhan SARS-CoV-2 RBD-Fc sample is usually compared against the reference amino acid sequence of Wuhan strain only (Fig. 2). Identity of Wuhan strain might be confirmed but potential contaminants from other strains would not be identified. By having the peptide key alongside the reference of target protein, purity of intermediate substances and vaccine products can be more ascertained. Generally, the limit of LC–MS detection is in a range of 1–10 ng ml^−1^ solution or ng mg^−1^ protein^33,34^. Within this study, a protein spike-in assay was performed to examine the limits of peptide detection. Among nine characteristic peptides, the lowest points of peptide detection at MS/MS level were around 5 ng ml^−1^, observed from peptide 5 and 8 (Table 1). These two peptides are unique for detecting RBD-Fc derived from Lambda, Zeta, Epsilon or Kappa strains. Peptide 3 for detecting RBD-Fc derived Gamma strain and peptide 6.1 for detecting RBD-Fc derived from Iota strain were detectable at concentration around 20 ng ml^−1^, demonstrating relatively sensitive detection by this developed LC–MS method. Detection limits of other peptides were in a range of 50–200 ng ml^−1^, which would be sufficient for detecting even small contaminations in our vaccine production. Contaminants from other strains likely occur at microgram level since the tested materials are typically prepared at 1 mg ml^−1^.

According to the WHO guideline, cross-contamination of biological materials at any stage during biopharmaceutical manufacturing is a risk and must be assessed and controlled^19^. In our production system, biological materials can be derived from different SARS-CoV-2 strains, therefore appropriate logistic plans and activities, such as a clear separation of storage areas between different bacteria cell banks and avoiding infiltrating different strains at the same time, must be exercised to reduce the risk. These processes have already been implemented in the manufacturing plant of Baiya Phytopharm. An implementation of LC–MS analysis in the QC process will ensure the purity of starting materials and final vaccine products. Furthermore, molecular techniques, such as real-time PCR, could be further developed to check contaminations of different SARS-CoV-2 strains in bacterial clones and DNA plasmids during cloning and transformation steps. It could also be used to track down microbial contaminations and host cell DNA residues in the final vaccine products^35^. By using bioanalytical and molecular techniques in combination, QC analysis in biologic manufacture would be more forceful to ensure production integrity and product quality.

## Conclusion

The LC–MS approach was developed to confirm the RBD sequence of Wuhan wild type strain in SARS-CoV-2 RBD-Fc vaccine production and to detect contaminations of other SARS-CoV-2 strains in the products. The method was incorporated into our QC procedures to ensure product quality. The generated peptide key, comprising nine characteristic peptides, facilitates SARS-CoV2 strain identification. All 14 SARS-CoV-2 strains, except Kappa and Epsilon, can be clearly distinguished in one step of detection. GluC enzyme could be further applied to facilitate the differentiation of RBD-Fc derived from Kappa and Epsilon strains. The limit of LC–MS detection likely covers the range of protein contaminations in drug substances and final vaccine products. This method would not only improve QC system in our vaccine production but can be developed for characterizing SARS-CoV-2 variants in other clinical or virological COVID-19-related research.

## Methods

### Plant materials

Original seeds of *Nicotiana benthamiana* plant used in this study were supported by Dr. Supaart Sirikantaramas, Faculty of Science, Chulalongkorn University. Plants were grown in a closed room under controlled conditions at Faculty of Pharmaceutical Sciences, Chulalongkorn University with permission from the Institutional Biosafety Committee of Chulalongkorn University (CU-IBC). Plant material collection and waste management complied with the safety guidelines regulated by the Center for Safety, Health and Environment of Chulalongkorn University (SHECU).

### Construction of SARS-CoV-2 RBDs in conjugation with Fc region of IgG1

Nucleotide sequences of the RBD region of various SARS-CoV-2 strains, including Wuhan wild-type, Alpha, Beta, Gamma, Delta, Epsilon, Zeta, Eta, Theta, Iota, Kappa, Lambda, Omicron BA1 and Omicron BA2 were obtained from the NCBI database and synthesized using an oligo synthesizer. The RBD sequences were fused with the sequences of human Fc region immunoglobulin G1 (IgG1) using 3 sets of GGGGS linker. The KDEL sequence was added at C-terminus of Fc region to establish high-mannose *N*-glycan patterns^36^. Recombinant DNA was ligated into a geminiviral vector, pBaiya, and transformed into *Agrobacterium tumefaciens* GV3101 using electroporation method^37^. A graphic illustration showing amino acid differences within the RBD region among all SAR-CoV-2 strains is depicted in Fig. 1.

### Transient expression of RBD-Fc fusion protein in *N. benthamiana*

Infiltration steps were performed as described previously^38,39^. Briefly, *A. tumefaciens*, containing the pBaiya vector inserted with SARS-CoV-2 RBD-Fc IgG1 recombinant protein, was grown at 28 °C for two days and infiltrated into three-week-old hydroponically grown *N. benthamiana* host plants using a vacuum chamber. Infected plants were maintained at 28 °C with 16 h light and 8 h darkness for three days when wilting symptoms were observed.

### Protein purification

Plant leaves were manually harvested and extracted using a 1 × phosphate buffer saline (PBS) buffer. Leaf extracts were initially filtered using a filter cloth and then centrifuged at 26,000×*g* for 40 min at 4 °C. Supernatant was filtered again through 0.45 µm membrane filter using vacuum filtration and loaded onto protein A column. The column was washed with 1 × PBS and the protein was eluted using 0.1 M glycine buffer, pH 2.9. Protein solution was neutralized with 1 M Tris–HCl, pH 8.8 and then dialyzed against 1 × PBS (cell grade) for three cycles. The dialyzed protein was concentrated using Amicon Ultra-4 30 kDa molecular weight cutoff (MWCO) centrifugal device. Protein concentration was measured using Bradford assay and adjusted to a final concentration of 1 mg ml^−1^.

### Tryptic digestion

Approximately 20 µg of plant-produced SARS-CoV-2 RBD-Fc proteins of all strains was desalted and buffer exchanged to 100 µl of 50 mM ammonium bicarbonate (ABC) buffer using Pall NanoSep 30 kDa MWCO centrifugal device. Final protein concentration was approximately 200 µg ml^−1^. Protein solution was reduced with 10 mM dithiothreitol (DTT) at 65 °C for 30 min and alkylated with 25 mM iodoacetamide (IAA) at room temperature for 20 min under darkness. Proteins were digested with 0.5 µg trypsin at 37 °C for 4 h. The reaction was stopped using 10% formic acid (FA) and centrifuged at 14,000 rpm for 10 min. Supernatant was transferred to a polypropylene vial and subjected to LC–MS/MS analysis.

### LC–MS/MS acquisition

Peptide samples were analyzed using Agilent 1290 Infinity II LC system coupled with Agilent 6545XT Q-TOF mass spectrometer. LC separation was conducted on AdvanceBio Peptide Mapping column (120 Å, 2.1 × 150 mm, 2.7 µm) at 60 °C. Injection volume was 10 µl. Mobile phase A was 0.1% FA in water and mobile phase B was 0.1% FA in acetonitrile. LC gradient was set as follows; 0% B for 2 min, 0–20% B in 33 min, 20–30% B in 20 min, 30–50% B in 10 min, 50–90% B in 5 min, 90% B for 5 min, 90–0% B in 5 min and 0% B for 5 min, with constant flow rate of 0.4 ml min^−1^. MS analysis was conducted in positive mode with a mass range of 100–1700 m/z. MS parameters were set as follows; gas temperature at 325 °C, nebulizer at 35 psi, dying gas at 13 L min^−1^, sheath gas temperature at 350 °C, sheath gas flow at 12 L min^−1^, capillary voltage at 4000 V, nozzle voltage at 500 V, fragmentor voltage at 175 V and skimmer voltage at 65 V. Acquisition time was 1 spectrum per s. Maximum 2 precursor ions per cycle were selected for MS/MS fragmentation. Collision energy (CE) was varied according to the charge state of the peptide. For peptides with charge + 1 and + 2, the CE was calculated using a formula of (3.1 × ((m/z)/100) + 1), while peptides with charge ≥  + 3, the CE was calculated using a formula of (3.6 × ((m/z)/100) − 4.8).

### Data analysis

Raw data was uploaded to Agilent MassHunter BioConfirm software version 11.0. The data was processed using peptide mapping workflow. Total ion chromatogram was integrated and extracted to obtain MS spectrum using default settings. The resulting peptides were matched against the reference SARS-CoV-2 protein sequences with MS mass error at ± 10 ppm and MS/MS mass error at ± 30 ppm. False discovery rate (FDR) was set at 1%. Only peptides with MS/MS fragmentations were considered for protein identification. Trypsin was selected as a digestion method. Cysteine carbamidomethylation was a fixed modification. Phosphorylation and oxidation were selected as variable modifications. Matched peptides were used to confirm RBD sequence of Wuhan SARS-CoV-2 strain (Fig. 2) and to create the peptide key for detecting contaminations derived from RBD-Fc of other strains (Fig. 3).

### Determination of detection limit of each characteristic peptide

Wuhan RBD-Fc proteins were mixed with the proteins derived from other 13 SARS-CoV-2 strains, including Alpha, Beta, Gamma, Delta, Epsilon, Zeta, Eta, Theta, Iota, Kappa, Lambda, Omicron BA1 and Omicron BA2 in a final concentration of 200 µg ml^−1^. Protein mixture was diluted to 10 different concentrations, including 100, 50, 20, 10, 5, 2, 1, 0.5, 0.2 and 0.1 µg ml^−1^. The samples were tryptic digested and processed to LC–MS analysis according to the method described above. The lowest dilution that the peptide was detected in MS and MS/MS modes is reported. In addition, peak areas of the peptide detected in MS mode were plotted against protein concentrations. The detection limit was calculated using the formula of (3.3 × standard deviation of the response) / slope of the calibration curve.

### Supplementary Information

Supplementary Information.

## Data Availability

All data generated in this study is supplied within this article and supplementary materials.
