# Supplementary material for: Quality control in SARS-CoV-2 RBD-Fc vaccine production using LC–MS to confirm strain selection and detect contaminations from other strains
Source: Sci Rep. 2024 Apr 26;14:9629. doi: 10.1038/s41598-024-59860-4 (PMC11053075; doi:10.1038/s41598-024-59860-4)
Supplement: Supplementary file 1 — Supplementary Information. [file 41598_2024_59860_MOESM1_ESM.docx]

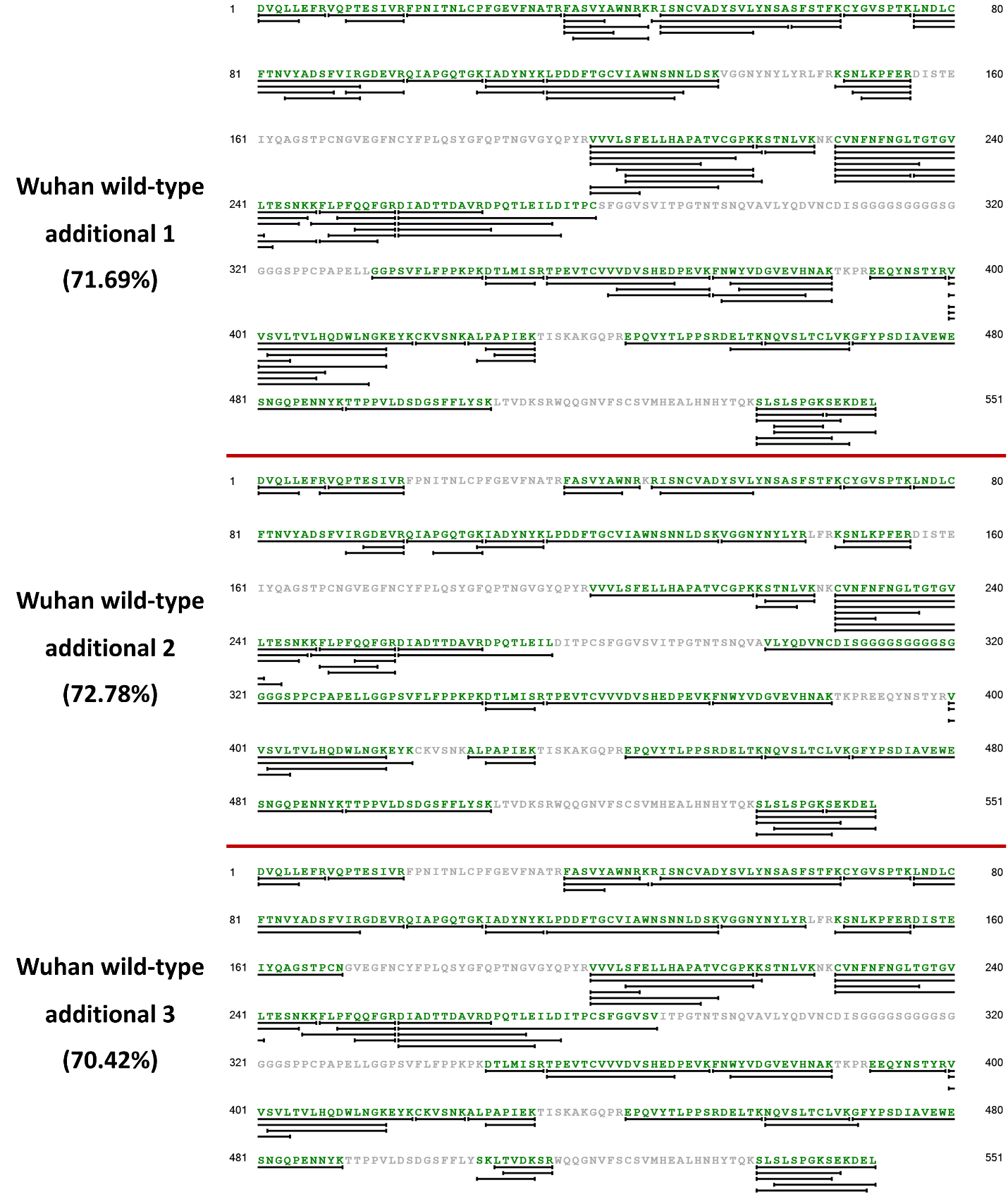


**Supplementary Fig. S1.** Confirmation of Wuhan RBD-Fc IgG1 protein in other acquisitions. Peptides detected with MS/MS fragmentation are highlighted in green. Black lines underneath amino acid sequences demonstrate peptide coverage. Overall sequence coverages of additional runs 1 to 3 were 71.69%, 72.78% and 70.42%, respectively.


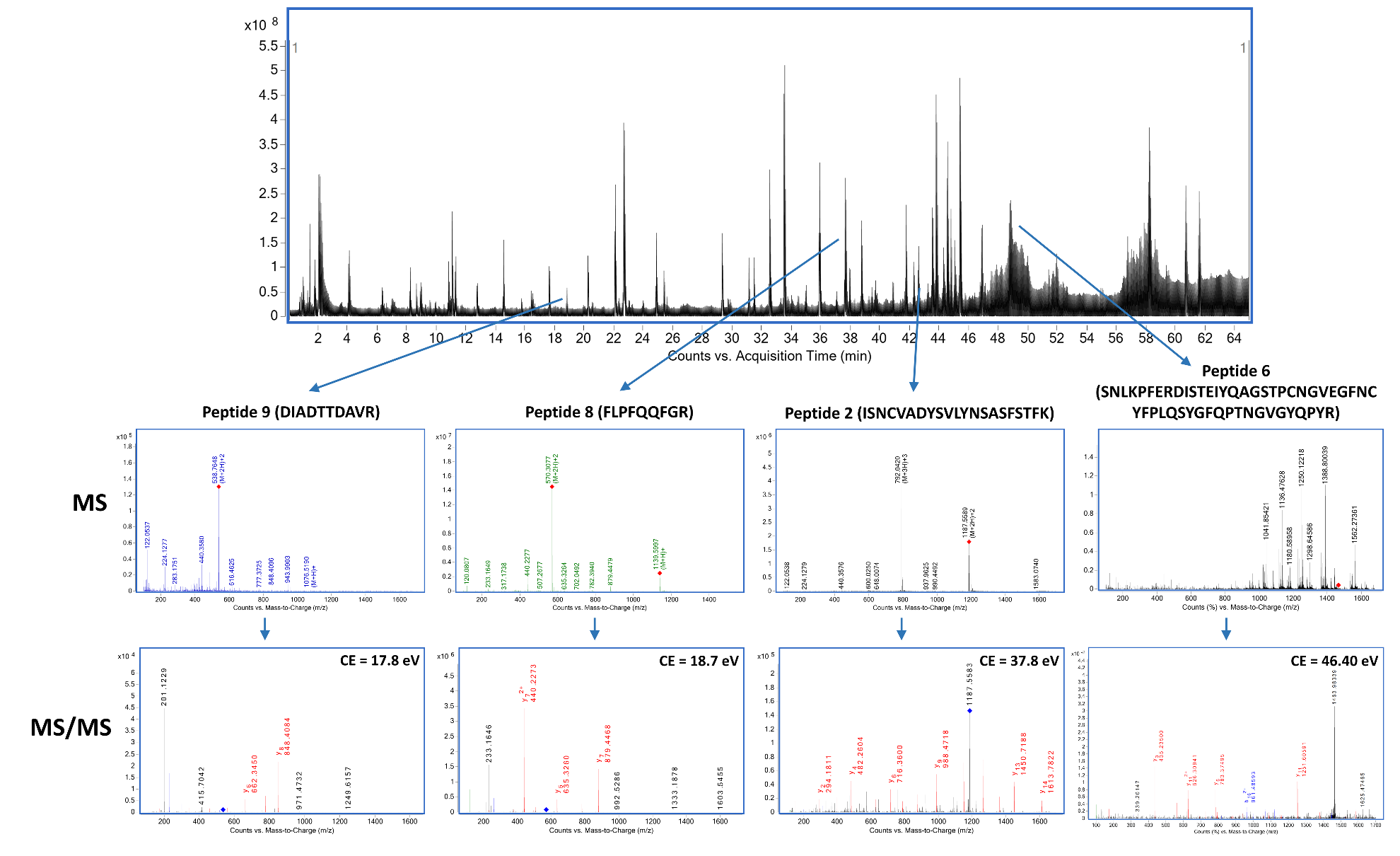


**Supplementary Fig. S2.** LC chromatogram and representative MS and MS/MS spectrum of peptides 2, 6, 8 and 9 upon the identification of recombinant Wuhan SARS-CoV-2 RBD-Fc protein sample.

**Supplementary Table S1.** Details of characteristic peptides used for SARS-CoV-2 strain identification in this analysis**.**

| **Peptide** | **SARS-CoV-2 strains** | **Retention time (min)** | **m/z** | **Mass (Da)** | **Diff (ppm)** | **Modification** | **Collision energy (eV)** | **MS/MS score (%)** |
| --- | --- | --- | --- | --- | --- | --- | --- | --- |
| **Peptide 1** | | | | | | | | |
| FPNITNLCPFGEVFNATR | Wuhan or others | 52.2 | 1,046.0127 | 2,096.0119 | -1.43 | Iodoacetamide | 33.6 | 93.25 |
| FPNITNLCPFDEVFNATR | Omicron BA1 or BA2 | 52.2 | 1,078.0187 | 2,154.0240 | 1.66 | Iodoacetamide | 34.5 | 100.00 |
| **Peptide 2** | | | | | | | | |
| ISNCVADYSVLYNSASFSTFK | Wuhan or others | 42.6 | 791.7076 | 2,372.1010 | 0.65 | Iodoacetamide | 37.8 | 100.00 |
| ISNCVADYSVLYNLAPFFTFK | Omicron BA1 | 59.2 | 823.7428 | 2,468.2058 | -1.13 | Iodoacetamide | 39.3 | 84.14 |
| ISNCVADYSVLYNFAPFFAFK | Omicron BA2 | 59.2 | 825.0682 | 2,472.1820 | -0.16 | Iodoacetamide | 39.4 | 93.91 |
| **Peptide 3** | | | | | | | | |
| QIAPGQTGK | Wuhan or others | 11.0 | 450.2552 | 898.4932 | 1.96 | None | 15.0 | 86.57 |
| GNEVSQIAPGQTGNIADYNYK | Omicron BA2 | 29.5 | 1,120.0373 | 2,238.0622 | 3.07 | None | 35.8 | 89.40 |
| QIAPGQTGNIADYNYK | Omicron BA1 or Beta | 26.3 | 876.9307 | 1,751.8473 | -0.35 | None | 28.2 | 93.97 |
| QIAPGQTGTIADYNYK | Gamma | 26.3 | 870.4331 | 1,738.8527 | 0.02 | None | 28.0 | 100.00 |
| **Peptide 4** | | | | | | | | |
| LPDDFTGCVIAWNSNNLDSK | Wuhan or others | 40.7 | 1,133.5261 | 2,265.0379 | 0.31 | Iodoacetamide | 36.2 | 100.00 |
| LPDDFTGCVIAWNSNKLDSK | Omicron BA1 or BA2 | 40.7 | 1,133.5240 | 2,265.0357 | -0.67 | Iodoacetamide | 36.2 | 100.00 |
| **Peptide 5** | | | | | | | | |
| VGGNYNYLYR | Wuhan or others | 24.9 | 609.7987 | 1,217.5827 | -0.22 | None | 20.0 | 89.24 |
| VSGNYNYLYR | Omicron BA1 | 24.4 | 624.8037 | 1,247.5929 | -0.51 | None | 20.4 | 89.01 |
| VGGNYNYQYR | Lambda | 17.9 | 617.2857 | 1,232.5570 | -0.34 | None | 20.1 | 78.09 |
| VGGNYNYR | Delta, Kappa or Epsilon | 13.6 | 471.7250 | 941.4352 | -0.33 | None | 15.7 | 85.76 |
| **Peptide 6.1** | | | | | | | | |
| DISTEIYQAGSTPCN | Wuhan, Alpha, Epsilon, Kappa or Lambda | 28.2 | 828.3635 | 1,654.7123 | -1.32 | Iodoacetamide | 26.7 | 83.66 |
| DISTEIYQAGSK | Delta | 25.9 | 656.3247 | 1,310.6347 | -0.52 | None | 21.4 | 86.84 |
| DISTEIYQAGNTPCNGVK | Iota | 28.9 | 656.3112 | 1,965.9118 | 0.81 | Iodoacetamide | 31.5 | 100.00 |
| DISTEIYQAGSTPCNGVK | Beta, Gamma, Zeta, Theta or Eta | 29.3 | 647.3070 | 1,938.8987 | -0.31 | Iodoacetamide | 31.1 | 100.00 |
| DISTEIYQAGNKPCNGVAGFNCYFPLR | Omicron BA1 or BA2 | 44.1 | 773.6163 | 3090.4347 | 0.62 | Iodoacetamide (×2) | 50.9 | 100.00 |
| **Peptide 6.2** | | | | | | | | |
| GFNCYFPLQSYGFQPTYGVGYQPYR | Beta, Gamma, Theta | 48.7 | 1,003.7938 | 3,008.3596 | 0.14 | Iodoacetamide | 47.7 | 87.66 |
| GFNCYFPLQSYGFQPTNGVGYQPYR | Zeta, Eta, Iota | 46.5 | 987.4539 | 2,959.3398 | 0.32 | Iodoacetamide | 46.9 | 79.33 |
| **Peptide 7** | | | | | | | | |
| CVNFNFNGLTGTGVLTESNKK | Wuhan or others | 38.7 | 767.3841 | 2,299.1307 | 1.76 | Iodoacetamide | 36.7 | 100.00 |
| CVNFNFNGLK | Omicron BA1 | 33.4 | 606.7947 | 1,211.5748 | -0.76 | Iodoacetamide | 19.9 | 86.88 |
| **Peptide 8** | | | | | | | | |
| FLPFQQFGR | Wuhan or others | 37.4 | 570.3045 | 1,138.5940 | 1.44 | None | 18.7 | 87.57 |
| FLPFQQLGR | Zeta | 36.5 | 553.3122 | 1,104.6097 | 1.56 | None | 18.2 | 87.59 |
| **Peptide 9** | | | | | | | | |
| DIADTTDAVR | Wuhan or others | 19.2 | 538.7648 | 1,075.5150 | 0.43 | None | 17.8 | 86.50 |
| DIDDTTDAVR | Alpha | 17.6 | 560.7593 | 1,119.5038 | -0.51 | None | 18.4 | 76.91 |
